# Long‐term antithrombotic management patterns in Asian patients with acute coronary syndrome: 2‐year observations from the EPICOR Asia study

**DOI:** 10.1002/clc.23400

**Published:** 2020-07-02

**Authors:** Bo Zheng, Yong Huo, Stephen W.‐L. Lee, Jitendra P. S. Sawhney, Hyo‐Soo Kim, Rungroj Krittayaphong, Stuart J. Pocock, Vo T. Nhan, Angeles Alonso Garcia, Chee Tang Chin, Jie Jiang, Stephen Jan, Ana Maria Vega, Nobuya Hayashi, Tiong K. Ong

**Affiliations:** ^1^ Department of Cardiology Peking University First Hospital Beijing China; ^2^ Department of Medicine Queen Mary Hospital Hong Kong SAR China; ^3^ Department of Cardiology Sir Ganga Ram Hospital New Delhi India; ^4^ Division of Cardiology, Department of Internal Medicine Seoul National University Hospital Seoul South Korea; ^5^ Division of Cardiology, Department of Medicine Faculty of Medicine, Siriraj Hospital Bangkok Thailand; ^6^ Department of Medical Statistics London School of Hygiene and Tropical Medicine London UK; ^7^ Department of Medicine Cho Ray Hospital Ho Chi Minh City Vietnam; ^8^ Imperial College, National Health Service (NHS) Trust London UK; ^9^ Department of Cardiology National Heart Centre Singapore Singapore; ^10^ Health Economics Program, The George Institute for Global Health, University of New South Wales Sydney Australia; ^11^ Observational Research Centre, Global Medical Affairs AstraZeneca, Madrid Spain; ^12^ Department of Biometrics AstraZeneca K.K Osaka Japan; ^13^ Department of Cardiology Sarawak General Hospital Kuching Malaysia

**Keywords:** acute coronary care antithrombotic management patterns, acute coronary syndrome, EPICOR, observational

## Abstract

**Background:**

Despite guideline recommendations, dual antiplatelet therapy (DAPT) is frequently used for longer than 1 year after an acute coronary syndrome (ACS) event. In Asia, information on antithrombotic management patterns (AMPs), including DAPT post discharge, is sparse. This analysis evaluated real‐world AMPs up to 2 years post discharge for ACS.

**Hypothesis:**

There is wide variability in AMP use for ACS management in Asia.

**Methods:**

EPICOR Asia (NCT01361386) is a prospective observational study of patients discharged after hospitalization for an ACS in eight countries/regions in Asia, followed up for 2 years. Here, we describe AMPs used and present an exploratory analysis of characteristics and outcomes in patients who received DAPT for ≤12 months post discharge compared with >12 months.

**Results:**

Data were available for 12 922 patients; of 11 639 patients discharged on DAPT, 2364 (20.3%) received DAPT for ≤12 months and 9275 (79.7%) for >12 months, with approximately 60% still on DAPT at 2 years. Patients who received DAPT for >12 months were more likely to be younger, obese, lower Killip class, resident in India (vs China), and to have received invasive reperfusion. Clinical event rates during year 2 of follow‐up were lower in patients with DAPT >12 vs ≤12 months, but no causal association can be implied in this non‐randomized study.

**Conclusions:**

Most ACS patients remained on DAPT up to 1 year, in accordance with current guidelines, and over half remained on DAPT at 2 years post discharge. Patients not on DAPT at 12 months are a higher risk group requiring careful monitoring.

## 1 INTRODUCTION

Acute coronary syndromes (ACS) are a leading cause of morbidity and mortality in the Asia‐Pacific (APAC) region.^1^, ^2^ Although APAC guidelines are generally comparable to those from Europe and the United States, a higher proportion of patients in the region receive solely medical management after hospitalization for an ACS, as opposed to invasive procedures. Post‐discharge medical management can vary considerably across Asia, as well as ACS‐related outcomes, which are, overall, poorer in the APAC region when compared with global outcomes.^1^, ^2^, ^3^ The relationship between patterns of medical management post‐ACS discharge and ACS‐related outcomes in Asia remains unclear.

The pivotal CHARISMA (Clopidogrel for High atherotherombotic Risk and Ischemic Stablization, Management, and Avoidance) trial demonstrated that dual antiplatelet therapy (DAPT) with clopidogrel plus aspirin for a median of 28 months was effective in reducing a composite endpoint (myocardial infarction [MI], stroke, or death from cardiovascular causes) compared with aspirin alone in patients with clinically evident atherothrombosis, but not the overall population with either clinically evident cardiovascular disease or multiple risk factors.^4^ A subsequent subgroup analysis from CHARISMA confirmed the benefit of DAPT in patients with prior MI, ischemic stroke, or symptomatic peripheral arterial disease, with a significant reduction in the composite endpoint but no difference in severe bleeding compared with aspirin alone.^5^ As a result of this and subsequent studies, recent national and international guidelines recommend the use of DAPT for patients surviving an ACS, including those with ST‐elevation myocardial infarction (STEMI) or non‐ST‐elevation ACS (NSTE‐ACS), the latter comprising non‐ST‐elevation myocardial infarction (NSTEMI) and unstable angina (UA).^6^, ^7^, ^8^, ^9^, ^10^, ^11^, ^12^ In general, guidelines recommend the use of DAPT for up to 12 months post‐ACS, with a switch to single antiplatelet therapy (SAPT) as appropriate, if bleeding risk is increased. However, guidelines are emerging that support prolonged DAPT in patients with ACS deemed to be at high risk of further ischemic events, particularly where the risk of ischemic events and cardiovascular death outweigh the risk of life‐threatening bleeding.^12^, ^13^, ^14^

A recent real‐world observational study showed that the proportion of patients receiving DAPT for more than a year after discharge post MI was higher in the APAC region (39%) than in Europe (12%),^15^ but data describing the impact of prolonged DAPT on outcomes in Asia are limited. The real‐world EPICOR Asia (long‐tErm follow‐uP of antithrombotic management patterns In acute CORonary syndrome patients in Asia) study (NCT01361386) followed ACS survivors across eight countries and regions in Asia for up to 2 years after an ACS event.^3^ EPICOR Asia had two co‐primary outcomes analyses performed; the first, reported elsewhere, examined the relationship between patient characteristics and long‐term outcomes according to index event diagnosis.^16^ The second, reported here, examined the relationship between patient characteristics and antithrombotic management patterns (AMPs) used, and observed long‐term clinical outcomes. Economic outcomes have also been examined.^17^, ^18^

## 2 METHODS

### 2.1 Study design and patients

Details of the EPICOR Asia study design and baseline patient characteristics have been reported previously.^3^ EPICOR Asia was a multinational, prospective, observational cohort study with a 2‐year post‐discharge follow‐up period, which described both in‐hospital and post‐discharge AMPs in ACS and associated clinical outcomes in eight countries and regions in Asia. Between June 2011 and May 2012, patients surviving initial hospitalization for ACS were enrolled from 218 centers in China, Hong Kong, India, Malaysia, Singapore, South Korea, Thailand, and Vietnam. Adults aged ≥18 years were enrolled if hospitalized for an ACS event within 48 h of symptom onset (STEMI, NSTEMI, or UA) and survived to discharge.

Overall, 13 005 patients were enrolled in EPICOR Asia, most from China (63.6%) and India (19.1%). Among these, 12 922 were eligible for inclusion; 83 patients were excluded (19 did not survive to discharge, ie, were incorrectly enrolled, and 64 were excluded—from one site—due to critical data quality issues). Data on patient demographics, medical history, and management were collected from symptom onset to discharge and AMPs at hospital discharge and post discharge by means of telephone interviews scheduled at 6 weeks and then every 3 months post index event until 2 years.

Study procedures adhered to International Conference on Harmonization (ICH) Good Clinical Practice guidance, the Declaration of Helsinki, and local regulations, and Institutional Review Board/Ethics Committee approval was obtained at each participating center in each country (https://astrazenecagrouptrials.pharmacm.com/ST/Submission/Search). All patients provided written informed consent.

The aim of this analysis was to characterize oral AMPs (antiplatelet and anticoagulant) at discharge and during a 2‐year follow‐up period. Patterns of antiplatelet therapy (none, aspirin only, P2Y_12_ receptor antagonist only [eg, clopidogrel or prasugrel], other SAPT, DAPT with aspirin plus P2Y_12_ receptor antagonist, other DAPT, or triple antiplatelet therapy [TAPT]), and anticoagulant (anticoagulant alone or anticoagulant plus antiplatelet therapy) were evaluated at discharge, and at 1.5, 3, 6, 9, 12, 18, and 23 months post discharge. In practice, final interviews were conducted within ±2 weeks of the 24‐month timepoint, hereafter referred to as 24 months or 2 years. Where appropriate, to avoid “loss” of patients whose last interview was 1 or 2 weeks prior to the 24‐month timepoint, medication status at 23 months is reported. For patients on SAPT or DAPT at discharge, the proportions remaining on SAPT or DAPT, or who switched to another antiplatelet pattern, were evaluated. Possible associations between baseline factors, SAPT use at discharge, and DAPT duration >12 months vs ≤12 months were investigated.

Time from discharge to event (including time to first major bleeding event) was assessed, including analysis by EPICOR 2‐year risk score, based on previously published data from the EPICOR^19^ and EPICOR Asia studies.^20^ Clinical events were death and the composite of death, non‐fatal MI, or non‐fatal ischemic stroke (with or without major bleeding).

All outcomes were validated as previously described.^3^

### 2.2 Statistical analysis

Patient characteristics are described using appropriate descriptive statistics, with *P* values derived from chi‐square test for categorical variables, two‐sample *t*‐test for comparison of means, and Wilcoxon test for comparison of medians. Logistic regression models were used to evaluate relationships between baseline characteristics, and (a) SAPT use at discharge, and (b) DAPT use >12 vs ≤12 months, with odds ratio (OR) and two‐sided 95% confidence interval (CI) presented. A stepwise model selection was made with the *P* value cut‐offs for selection at *P* < .01 and retention at *P* < .20.

Data are presented in terms of OR and associated two‐sided 95% CIs and *P* values. The proportion of patients discharged on DAPT who remained on DAPT beyond 12 months is also described by percentile (≤60th, >60th and ≤80th, >80th and ≤90th, and >90th, referred to as low, moderate, high, and very high risk categories, respectively) for each EPICOR 2‐year risk score category.

An exploratory analysis of time from 12 months after discharge to each clinical event during the second year of follow‐up by DAPT duration (>12 or ≤ 12 months) was made based on Cox proportional hazards models with stepwise model selection with DAPT duration (>12 or ≤12 months), index event diagnosis, age (<60, 60 to <75, and ≥75 years), gender, country group (defined as: China; India; Hong Kong, Singapore, South Korea [HK/Si/SK]; and Malaysia, Thailand, and Vietnam [Ma/Th/Vie]), and in‐hospital management always kept in the model. The data are presented in terms of hazard ratio (HR) and two‐sided 95% CIs.

Statistical analysis was performed using SAS version 9.3 or later (SAS Institute Inc., Care, North Carolina).

## 3 RESULTS

### 3.1 Patient characteristics

Details of baseline patient characteristics have been published previously.^3^ Of 12 922 patients enrolled in EPICOR Asia, most were from China (63.6%) and India (19.1%). Patients had a mean age of 60 years, 76.3% were male, 27.2% had prior cardiovascular disease (CVD), and 80.5% had cardiovascular risk factors (53.0% hypertension, 17.0% hypercholesterolemia, 24.5% diabetes, 8.4% family history of coronary artery disease [CAD], 5.3% obesity, and 33.9% current smokers). The final diagnosis was STEMI in 51.2%, NSTEMI in 19.9%, and UA in 28.9% of patients. Patients from all countries showed a relatively high prevalence of cardiovascular risk factors, notably, obesity (body mass index [BMI] >30 kg/m^2^) in patients from Malaysia and Singapore. Most had undergone coronary revascularization, except those from India and Malaysia.

### 3.2 Antiplatelet therapy at discharge and follow‐up

**FIGURE 1:**
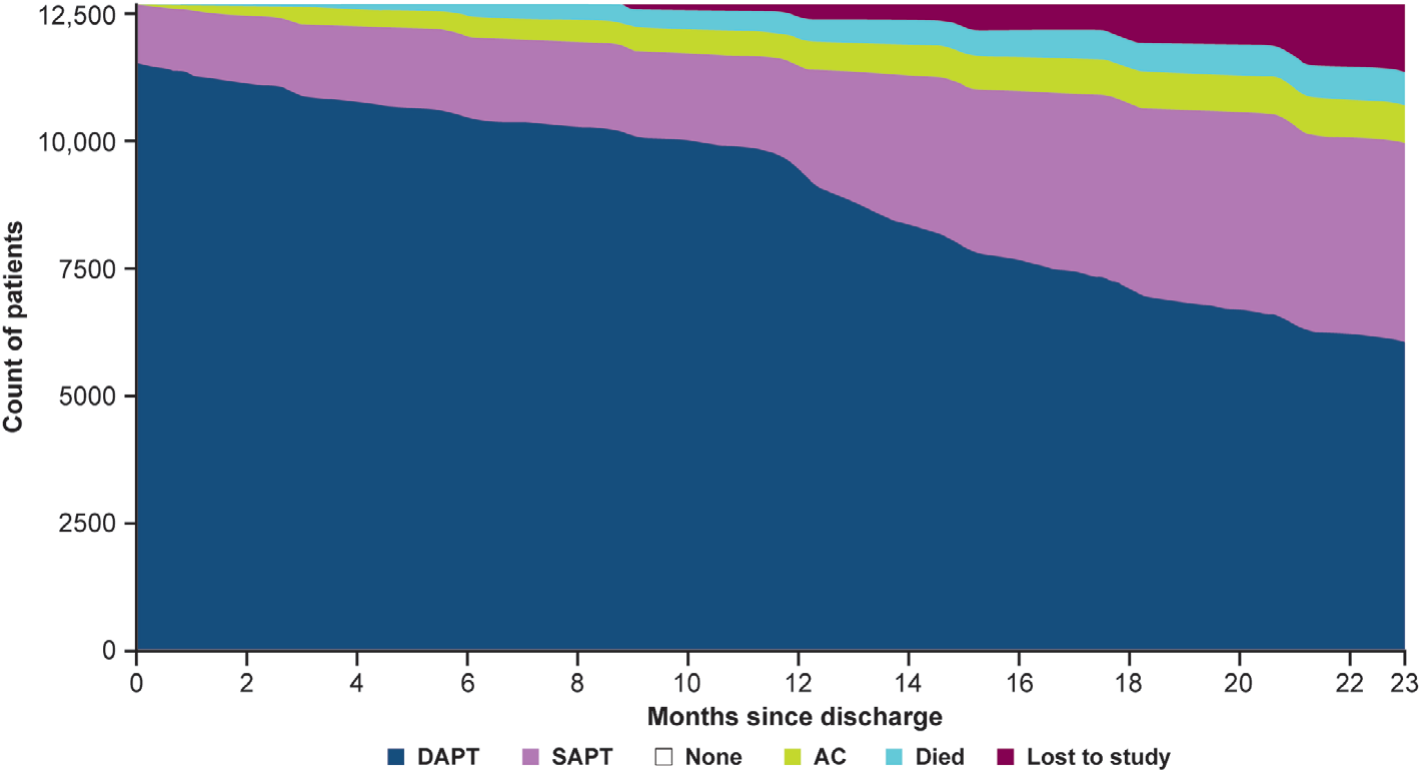
Antithrombotic management status of patients from discharge up to 2 years post discharge. Abbreviations: AC, anticoagulant; DAPT, dual antiplatelet therapy; SAPT, single antiplatelet therapy

Baseline demographics by discharge antiplatelet medication and by DAPT duration ≤12 or >12 months (in patients discharged on DAPT) are shown in Table 1 (patients who survived at least 12 months) and Supporting information, Table S1 (all patients), available in the Supporting information in the online version of this article. Of the 12 922 patients, 1283 (9.9%) were discharged on SAPT or no antiplatelet therapy, and 11 639 (90.1%) on DAPT (Supporting information, Table S2), consisting of aspirin and a P2Y_12_ receptor antagonist in 88.8% of cases (Supporting information, Table S3). By 12 months post discharge, 86.4% were still receiving DAPT, which decreased to 62.5% by 2 years (Figure 1). Aspirin was the most common antiplatelet prescribed at discharge as monotherapy, being used in 5.3% of patients, which increased to 31.2% at 2 years (Supporting information, Table S3). Patients discharged on DAPT were more likely to switch to SAPT (1.6%, 11.3%, and 32.5% at 1.5, 12, and 24 months, respectively), than to no therapy (0.4%, 2.2%, and 4.9%, respectively). Use of TAPT and/or an anticoagulant was minimal, with low usage throughout (Supporting information, Table S3).

**TABLE 1:** Demographics, index diagnosis, and general health status of patients discharged on DAPT who survived ≥12 months and continued on DAPT for ≤12 vs >12 months^a^

	Duration of DAPT following DAPT at discharge		
	≤12 months (n = 1542)	>12 months (n = 9275)	*P* value
Duration of DAPT^a^, months; median (IQR)	7.7 (3.0‐11.6)	23.6 (18.1‐23.8)	
Age group, years			.0001
≤59	716 (46.4)	4564 (50.2)	
60‐74	617 (40.0)	3681 (39.7)	
≥75	209 (13.6)	940 (10.1)	
Male	1149 (74.5)	7249 (78.2)	.002
Final diagnosis of index event			.046
STEMI	779 (50.5)	4940 (53.3)	
NSTE‐ACS	763 (49.5)	4335 (46.7)	
BMI^b^, mean (SD)	24.5 (3.3)	24.8 (3.6)	.002
BMI^b^			.26
<25 kg/m^2^ (underweight/normal)	867 (59.0)	4893 (56.9)	
25 to <30 kg/m^2^ (overweight)	524 (35.7)	3182 (37.0)	
≥30 kg/m^2^ (obese)	78 (5.3)	518 (6.0)	
Killip class			.02
I	787 (51.0)	4976 (53.7)	
II	158 (10.3)	983 (10.6)	
III	39 (2.5)	319 (3.4)	
IV	37 (2.4)	230 (2.5)	
Missing	521 (33.8)	2767 (29.8)	
Left bundle branch block	12 (0.8)	157 (1.8)	.009
Ejection fraction			.43
<30%	25 (1.6)	151 (1.6)	
30%‐40%	68 (4.4)	448 (4.8)	
≥40%	981 (63.6)	5699 (61.4)	
Missing	468 (30.4)	2977 (32.1)	
Any CVD risk factors^b^	1011 (66.0)	6072 (65.9)	.92
Hypertension	831 (54.3)	4859 (52.8)	.28
Hypercholesterolemia	273 (18.5)	1617 (18.1)	.72
Diabetes	335 (22.0)	2245 (24.5)	.032
Family history of CAD	146 (10.1)	798 (9.1)	.21
Current smoker	543 (35.2)	3700 (43.1)	.81
Any prior CVD^b^	439 (28.7)	2416 (26.7)	.09
MI	149 (9.8)	828 (9.2)	.42
Prior PCI	125 (8.2)	694 (7.7)	.46
Prior CABG	17 (1.1)	121 (1.3)	.49
CAG diagnostic for CAD	164 (10.8)	836 (9.3)	.06
Angina	247 (16.2)	1410 (15.6)	.53
Heart failure	37 (2.5)	194 (2.2)	.47
Atrial fibrillation	28 (1.9)	103 (1.1)	.022
TIA/stroke	67 (4.4)	386 (4.3)	.79
PVD	12 (0.8)	70 (0.8)	.93
Chronic renal failure	34 (2.3)	122 (1.4)	.007
Any CV medication^b^	584 (38.8)	3231 (36.5)	.08
Any antiplatelet	392 (26.3)	2097 (23.9)	.045
Aspirin	374 (25.1)	2028 (23.1)	.10
Clopidogrel	135 (9.1)	805 (9.2)	.90
β‐blocker	251 (17.6)	1327 (15.4)	.042
ACEi/ARB	234 (16.4)	1335 (15.5)	.43
Statin	241 (16.8)	1255 (14.6)	.031
In‐hospital procedures^b^			
PCI/CABG	932 (60.9)	7127 (78.1)	<.0001
Reperfusion (primary PCI or fibrinolysis)	998 (65.4)	7445 (81.5)	<.0001
Any drug‐eluting stent	797 (51.7)	6381 (68.8)	<.0001
In‐hospital MI/recurrent ischemia/heart failure/cardiogenic shock/arrhythmia^b^	266 (17.4)	1179 (12.9)	<.000
Country group^c^			<.0001
China (n = 7049)	1099 (71.3/15.6)	5950 (64.2/84.4)	
India (n = 1832)	140 (9.1/7.6)	1692 (18.2/92.4)	
South Korea, Hong Kong, Singapore (n = 847)	129 (8.4/15.2)	718 (7.7/84.8)	
Malaysia, Thailand, Vietnam (n = 1089)	174 (11.3/16.0)	915 (9.9/84.0)	
Time from symptom onset to admission, hours; median (IQR)	5.3 (2.2‐17.2)	5.7 (2.3‐18.0)	.19
Time from admission to reperfusion, hours; median (IQR)	10.3 (1.2‐97.3)	14.0 (1.4‐91.9)	.37
Time from symptom onset to reperfusion, hours; median (IQR)	24.0 (4.2‐112.7)	27.1 (5.7‐12.0)	.009
Length of hospital stay, days; median (IQR)	9.0 (6.0‐13.0)	8.0 (5.0‐12.0)	.004
Dependence at discharge			.05
No dependence	1471 (95.4)	8698 (93.8)	
Non‐severe dependence	66 (4.3)	537 (5.8)	
Severe dependence	5 (0.3)	40 (0.4)	
EQ‐5D overall health state at discharge, mean (SD)	79.2 (12.9)	78.9 (13.8)	.46

Abbreviations: ACEi/ARB, angiotensin‐converting enzyme inhibitor/angiotensin II receptor blocker; BMI, body mass index; CABG, coronary artery bypass graft; CAD, coronary artery disease; CAG, coronary angiogram; CHD, coronary heart disease; CV, cardiovascular; CVD, cardiovascular disease; DAPT, dual antiplatelet therapy; EQ‐5D, EuroQol‐5 Dimensions; IQR, interquartile range; MI, myocardial infarction; NSTE‐ACS, non‐ST elevation acute coronary syndrome; SD, standard deviation; STEMI, ST‐elevation myocardial infarction; PCI, percutaneous coronary intervention; TAPT, triple antiplatelet therapy; TIA, transient ischemic attack. Values are n (%) unless otherwise indicated.

a Includes patients reported as taking two or more antiplatelets at discharge (ie, including TAPT). DAPT duration was defined as time from discharge to the last use of two or more antiplatelets, not accounting for interruptions.

b At discharge, data were missing for 879 patients for BMI, 68 patients for any CVD risk factors, 244 patients for any prior CVD, 562 patients for any CV medication, 172 patients for in‐hospital PCI/CABG, 169 patients for primary PCI/fibrinolysis, and 128 patients for in‐hospital MI/recurrent ischemia/heart failure/cardiogenic shock/arrhythmia.

c Percentages shown are within DAPT duration group/within each country or region.

Of those patients discharged on SAPT (n = 1121), 90.2% remained on SAPT at 12 months, and 85.4% at 2 years; these patients were more likely to switch to no antiplatelet (2.0%, 8.8%, and 13.6% at 1.5, 12, and 24 months, respectively) than to DAPT (0.5%, 1.0%, and 1.1%, respectively). No patient discharged on SAPT received TAPT post discharge.

Of the 11 817 patients discharged on DAPT who survived at least 12 months, 1542 received DAPT for ≤12 months (median 7.7 months) and 9275 for more than 12 months (median 23.6 months) (Table 1). Twelve‐month survivors who received DAPT for more than 12 months post discharge, compared with ≤12 months, were younger, had a higher BMI, and were more likely to be male and to have received an in‐hospital invasive procedure for the index event (Table 1 and Supporting information, Table S1). They were less likely to have received prior antiplatelet medication, experienced an in‐hospital cardiovascular event, had prior atrial fibrillation, or to have impaired renal function. Median time from symptom onset or admission to reperfusion (by intervention or fibrinolysis) was similar regardless of duration of DAPT use, whereas the overall time from symptom onset to reperfusion was shorter in the DAPT ≤12 months cohort (Table 1 and Supporting information, Table S1). Conversely, median length of hospital stay was shorter with DAPT >12 vs ≤12 months.

Multivariable analysis indicated that significant predictors of DAPT duration >12 vs ≤12 months in patients who survived at least 12 months were younger age, obesity, residency in India vs China, any drug‐eluting stent (DES) placement, and in‐hospital percutaneous coronary intervention (PCI)/coronary artery bypass graft (CABG) (Table 2). In contrast, patients who had an in‐hospital cardiovascular event were less likely to be on DAPT >12 months. The greatest predictor of SAPT/no antiplatelet vs DAPT at discharge was anticoagulant use (Supporting information, Table S4). Other significant predictors of SAPT/no antiplatelet use were any degree of physical dependence (vs no dependence), Killip class >I, female sex, and residency in India vs China, whereas patients with ejection fraction ≥30% and those who underwent in‐hospital intervention (PCI/CABG) were more likely to be discharged on DAPT.

**TABLE 2:** Logistic multivariable regression analysis for predictors of DAPT duration >12 months vs ≤12 months in patients who survived ≥12 months

Factor	OR^a^	95% CI	*P* value
Age group, vs ≤59 years			<.0001
60‐74	0.88	0.79, 0.98	
≥75	0.68	0.58, 0.79	
Bodyweight (BMI, kg/m^2^), vs <25 (underweight/normal)			<.05
Overweight (mean BMI, 25‐30 kg/m^2^)	1.13	1.02, 1.25	
Obese (BMI >30 kg/m^2^)	1.15	0.93, 1.43	
Any drug‐eluting stent, vs no	1.67	1.39, 2.00	<.0001
In‐hospital cardiovascular event, vs no	0.68	0.59, 0.77	<.0001
In‐hospital PCI/CABG, vs no	1.53	1.28, 1.85	<.0001
Country group, vs China			<.0001
India	1.75	1.50, 2.04	
Hong Kong, Singapore, and South Korea	1.05	0.87, 1.27	
Malaysia, Thailand, and Vietnam	1.27	1.08, 1.48	

Abbreviations: BMI, body mass index; CABG, coronary artery bypass graft; CI, confidence interval; DAPT, dual antiplatelet therapy; OR, odds ratio; PCI, percutaneous coronary intervention; TAPT, triple antiplatelet therapy. Stepwise selection procedure using logistic regression model for likelihood of DAPT duration >12 months (vs ≤12 months); DAPT duration was defined as time from discharge from the index hospitalization to the last use of ≥2 antiplatelets (ie, including TAPT), not accounting for interruptions. Continuous variables replaced with categorical equivalents included: BMI (<25, 25‐30, ≥30 kg/m^2^; retained) and initial creatinine (< and ≥1.2 mg/dL; not retained).

a OR > 1 indicates greater likelihood of DAPT duration >12 months.

**FIGURE 2:**
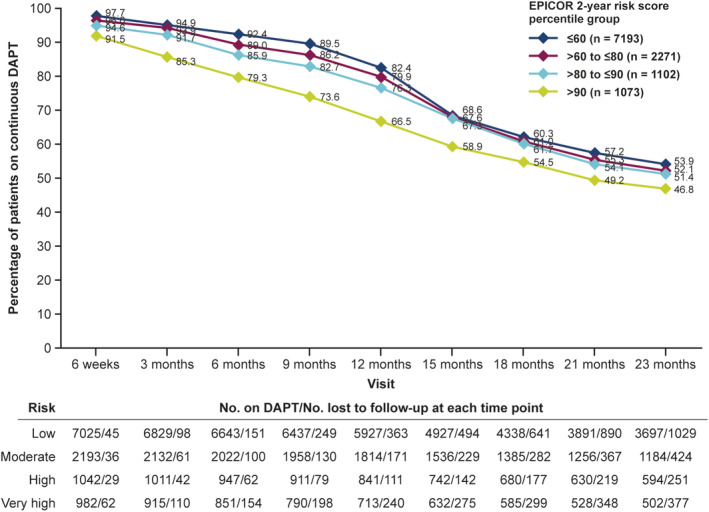
Proportion of patients on continuous DAPT at each visit according to EPICOR 2‐year risk categories^a^. Low risk, ≤60th percentile; moderate risk, >60th to ≤80th percentile; high risk, >80th to ≤90th percentile; and very high risk, >90th percentile of the EPICOR 2‐year risk score. Data at each time point are percentages of patients still on DAPT out of total discharged on DAPT in each risk category. ^a^Includes patients reported as taking two or more antiplatelets (ie, including triple antiplatelet therapy). Abbreviations: DAPT, dual antiplatelet therapy; EPICOR, long‐tErm follow‐up of antithrombotic management Patterns In acute CORonary syndrome

When evaluated in terms of EPICOR 2‐year risk score, the number of patients remaining on continuous DAPT were generally lower as risk percentiles increased, up to the 15‐month visit, although this pattern was less apparent thereafter (Figure 2; Supporting information, Table S5). There was greater loss to follow‐up at each timepoint in the highest percentile group (>90th).

**FIGURE 3:**
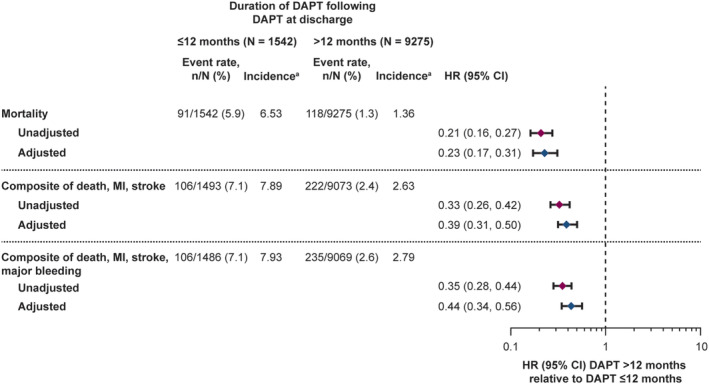
Hazard ratio between DAPT use >12 months and ≤12 months for clinical events during the second year of follow‐up in patients who survived ≥12 months. ^a^Number of (first) events per 100 patient‐years. Abbreviations: CI, confidence interval; DAPT, dual antiplatelet therapy; HR, hazard ratio; IS, ischemic stroke; MI, myocardial infarction

When comparing the groups who were treated with DAPT >12 months vs ≤12 months in the 12‐month survivors, the hazard of composite of death, MI, and stroke was much lower in the group treated with DAPT >12 months (adjusted HR 0.39) (Figure 3), seemingly lower than would be expected should treatment allocation bias and confounding have been sufficiently reduced. This was also apparent insofar as the adjusted HR was quite similar to the unadjusted HR (0.33). The same trend was shown for both mortality and when major bleeding was added to the above composite. Similarly, the incidence of each endpoint (number of first events per 100 patient‐years) was lower in the DAPT >12 months population.

## 4 DISCUSSION

The EPICOR Asia study revealed that, despite guideline recommendations at the time of the study, most patients with ACS in Asia remained on DAPT beyond 12 months. Logistic multivariable regression analysis indicated that these patients tended to be younger, obese, lower Killip class, resident in India (vs China), and to have received invasive reperfusion and DES placement, and they were less likely to have had an in‐hospital cardiovascular event. The approximate 60% observed rate of prolonged DAPT use (>12 months post‐discharge) in this study is generally higher than that reported across the APAC region (39%) and Europe (12%), albeit with considerable between‐country variability.^1^, ^2^, ^15^ Similarly, while 75% of the EPICOR Asia overall DAPT cohort underwent PCI/CABG for the index event, it has been reported elsewhere that up to 80% of patients with ACS in the APAC region are managed non‐invasively.^1^ In EPICOR Asia, event rates continued to accrue throughout the 2‐year follow‐up period post discharge, with higher rates in the initial period and a steady increase during subsequent months up to the end of follow‐up.

Overall, patients in whom DAPT was used for more than 12 months appeared to show lower rates of cardiovascular events and mortality compared with those in whom DAPT cessation occurred earlier, with no apparent increase in risk of major bleeding. However, any observed association between event rates and DAPT duration must be interpreted with caution, due to the possible presence of confounding factors. In particular, treatment allocation bias and confounding occurring 12 months post discharge, that were not explained by the discharge variables, could not be adjusted for.

A recent randomized, non‐inferiority study evaluating 6 months vs ≥12 months of DAPT following PCI with DES placement in 2712 South Korean patients with ACS (the SMART‐DATE [Safety of 6‐month Duration of Dual Antiplatelet Therapy After Acute Coronary Syndromes] study) observed a higher rate of MI in the short‐duration DAPT population over an 18‐month follow‐up period (1.8% vs 0.8%; HR 2.41, 95% CI 1.15‐5.05; *P* = .02).^21^ Although no significant differences were evident between groups in terms of outcomes, the authors concluded that short‐term DAPT may present risks in patients with ACS who have undergone current‐generation DES placement. In contrast, two meta‐analyses of randomized controlled trials, published in 2018, suggested that short‐term (≤6 months) DAPT post‐DES placement may provide benefit compared with DAPT up to 12 months towards reducing bleeding risk.^22^, ^23^ Potential benefit from short‐term DAPT after DES placement in older vs younger patients was also suggested.^23^ It should be noted, however, that these analyses included patients with both stable CAD and ACS. Another meta‐analysis of randomized trials found that DAPT duration over 1 year was associated with significant reductions in ischemic events (cardiovascular death, recurrent MI, and stroke) compared with aspirin alone in stabilized high‐risk patients with prior MI.^24^ Major bleeding was increased when DAPT was continued for more than a year but not fatal bleeding or non‐cardiovascular death.

Recently, using data from more than 23 000 patients included in both the EPICOR Asia and the EPICOR study (NCT01171404), characteristics and outcomes of medically managed patients with NSTE‐ACS and predictors of 1‐ and 2‐year post‐discharge mortality risk were identified.^20^, ^25^, ^26^ In this analysis, when evaluated in terms of EPICOR 2‐year risk score, the number of patients remaining on DAPT during follow‐up was generally reduced as risk percentiles increased, particularly up to the 15‐month visit.

Several potential limitations of the EPICOR Asia study include most patients being enrolled from China, resident in metropolitan areas, and enrolled at well‐equipped centers, thus not being fully representative of the region. Such considerations may partly explain discrepancies in these data and earlier reports for the APAC region; reliance on earlier data must factor in possible greater uptake of evidence‐based guidelines, specifically, the use of both DAPT and PCI, as evidenced in China.^27^ Also, it is possible that the exclusion of data for patients who did not survive to hospital discharge may have resulted in underestimation of overall mortality from ACS. It should also be emphasized again that a causal relationship between DAPT duration and clinical event rates in this analysis cannot be inferred, as the population was non‐randomized, with many possible reasons for discontinuing DAPT (eg, patients who stopped DAPT early may have had associated clinical comorbidities or socio‐economic issues). Also, as mentioned previously, there are potential confounding factors that we were not able to adjust for, and landmark analyses in a real‐would registry study may not account for survivor bias that might influence the final results. Finally, the study was carried out prior to the wide availability of the more potent oral antiplatelet agents, prasugrel and ticagrelor. The long‐term (>1 year) use of DAPT with ticagrelor plus aspirin has been shown to significantly reduce the risk of subsequent cardiovascular events in the PEGASUS‐TIMI 54 (Prevention of Cardiovascular Events in Patients with Prior Heart Attack Using Ticagrelor Compared to Placebo on a Background of Aspirin‐Thrombolysis in Myocardial Infarction 54) trial in patients with prior MI,^28^ and in the THEMIS (The Effect of Ticagrelor on Health Outcomes in Diabetes Mellitus Patients Intervention Study) trial in diabetic patients with stable coronary artery disease (without a history of MI or stroke),^29^ albeit with increased risk of major bleeding in each case. The THEMIS‐PCI study demonstrated a more favorable net clinical benefit with long‐term DAPT in diabetic patients with rather than without prior PCI.^30^ Recent real‐world data from the RENAMI (Registry of New Antiplatelets in patients with Myocardial Infarction) registry in 6 European countries also demonstrated that DAPT duration >1 year with either prasugrel or ticagrelor in propensity‐score matched ACS patients significantly reduced all‐cause death, cardiovascular death, and recurrent MI compared with DAPT <1 year after 20 months of follow‐up, but with increased risk of bleeding in women and older patients (>75 years).^31^

In summary, this analysis of results from EPICOR Asia has shown that most patients surviving an ACS in Asia were discharged on DAPT, and more than half remained on DAPT for up to 2 years of follow‐up. Prolonged DAPT was more likely to be employed in younger patients, and those with DES placement or in‐hospital PCI/CABG, and less likely to be used in China compared with India. Patients in whom DAPT treatment was stopped earlier than 12 months post discharge had higher rates of mortality and adverse cardiovascular events. While a causal relationship between outcomes and DAPT duration in this study cannot be inferred, the findings do suggest that patients who receive DAPT for ≤12 months are a high‐risk population and may require greater medical attention after discontinuation of DAPT than patients who receive DAPT for longer.

## CONFLICT OF INTEREST

B.Z., Y.H., S.W.L.L., J.P.S.S., H.S.K., A.A.G., J.J., and S.J. have nothing to disclose. R.K. has been a consultant or advisory board member for AstraZeneca and Boehringer Ingelheim. S.J.P. receives research funds from AstraZeneca. V.T.N. has received research grants from AstraZeneca, Servier, Sanofi, and Boston Scientific and has been a consultant or advisory board member for AstraZeneca, Pfizer, Sanofi, Boehringer Ingelheim, Servier, M.S.D., Abbott, Bayer, Novartis, Merck Serono, Biosensor, Biotronic, Boston Scientific, Terumo, and Medtronic. C.T.C. has received research support from Eli Lilly, honoraria from Medtronic, and has been a consultant or advisory board member for AstraZeneca. N.H. is an employee of AstraZeneca, and A.M.V. is a former employee of AstraZeneca. T.K.O. has acted as a consultant or advisory board member for Sanofi‐Aventis, Abbott Vascular, Boston Scientific, Boehringer Ingelheim, Novartis, and AstraZeneca.

## Supporting information


**SUPPLEMENTARY TABLE S1** Demographics, index diagnosis, and general health status of all patients discharged on DAPT who continued on DAPT for ≤12 vs >12 months^a^
**SUPPLEMENTARY TABLE S2** Demographics and baseline characteristics of patients discharged on SAPT/no antiplatelet or DAPT^a^
**SUPPLEMENTARY TABLE S3** Antithrombotic therapy at discharge and during follow‐up
**SUPPLEMENTARY TABLE S4** Logistic multivariable regression analysis for predictors of SAPT/no antiplatelet vs DAPT^a^ at discharge
**SUPPLEMENTARY TABLE S5** Patients on continuous DAPT at each visit according to EPICOR 2‐year risk percentiles^a^

## References

[1] Asia‐Pacific ACS Medical Management Working Group , Huo Y , Thompson P , et al. Challenges and solutions in medically managed ACS in the Asia‐Pacific region: expert recommendations from the Asia‐Pacific ACS Medical Management Working Group. Int J Cardiol. 2015;183:63‐75. 25662044 10.1016/j.ijcard.2014.11.195

[2] Chan MY , Du X , Eccleston D , et al. Acute coronary syndrome in the Asia‐Pacific region. Int J Cardiol. 2016;202:861‐869. 26476044 10.1016/j.ijcard.2015.04.073

[3] Huo Y , Lee SW , Sawhney JP , et al. Rationale, design, and baseline characteristics of the EPICOR Asia study (long‐tErm follow‐uP of antithrombotic management patterns In acute CORonary syndrome patients in Asia). Clin Cardiol. 2015;38(9):511‐519. 26206158 10.1002/clc.22431 PMC6490857

[4] Bhatt DL , Fox KA , Hacke W , et al. Clopidogrel and aspirin versus aspirin alone for the prevention of atherothrombotic events. N Engl J Med. 2006;354(16):1706‐1717. 16531616 10.1056/NEJMoa060989

[5] Bhatt DL , Flather MD , Hacke W , et al. Patients with prior myocardial infarction, stroke, or symptomatic peripheral arterial disease in the CHARISMA trial. J Am Coll Cardiol. 2007;49(19):1982‐1988. 17498584 10.1016/j.jacc.2007.03.025

[6] Amsterdam EA , Wenger NK , Brindis RG , et al. 2014 AHA/ACC guideline for the management of patients with non‐ST‐elevation acute coronary syndromes: a report of the American College of Cardiology/American Heart Association task force on practice guidelines. J Am Coll Cardiol. 2014;64(24):e139‐e228. 25260718 10.1016/j.jacc.2014.09.017

[7] O'Gara PT , Kushner FG , Ascheim DD , et al. 2013 ACCF/AHA guideline for the management of ST‐elevation myocardial infarction: a report of the American College of Cardiology Foundation/American Heart Association task force on practice guidelines. J Am Coll Cardiol. 2013;61(4):e78‐e140. 23256914 10.1016/j.jacc.2012.11.019

[8] Roffi M , Patrono C , Collet JP , et al. 2015 ESC guidelines for the management of acute coronary syndromes in patients presenting without persistent ST‐segment elevation: task force for the management of acute coronary syndromes in patients presenting without persistent ST‐segment elevation of the European Society of Cardiology (ESC). Eur Heart J. 2016;37(3):267‐315. 26320110 10.1093/eurheartj/ehv320

[9] Steg PG , James SK , Atar D , et al. ESC guidelines for the management of acute myocardial infarction in patients presenting with ST‐segment elevation. Eur Heart J. 2012;33(20):2569‐2619. 22922416 10.1093/eurheartj/ehs215

[10] Chinese Society of Cardiology of Chinese Medical Association, Editorial Board of Chinese Journal of Cardiology . Guideline and consensus for the management of patients with non‐ST‐elevation acute coronary syndrome (2016). Zhonghua Xin Xue Guan Bing Za Zhi. 2017;45(5):359‐376. 28511320 10.3760/cma.j.issn.0253-3758.2017.05.003

[11] China Society of Cardiology of Chinese Medical Association, Editorial Board of Chinese Journal of Cardiology . Guideline on the diagnosis and therapy of ST‐segment elevation myocardial infarction. Zhonghua Xin Xue Guan Bing Za Zhi. 2015;43(5):380‐393. 26419981

[12] Valgimigli M , Bueno H , Byrne RA , et al. 2017 ESC focused update on dual antiplatelet therapy in coronary artery disease developed in collaboration with EACTS: the task force for dual antiplatelet therapy in coronary artery disease of the European Society of Cardiology (ESC) and of the European Association for Cardio‐Thoracic Surgery (EACTS). Eur Heart J. 2018;39(3):213‐260. 28886622 10.1093/eurheartj/ehx419

[13] Parker WA , Storey RF . Long‐term antiplatelet therapy following myocardial infarction: implications of PEGASUS‐TIMI 54. Heart. 2016;102(10):783‐789. 26857211 10.1136/heartjnl-2015-307858

[14] Ibanez B , James S , Agewall S , et al. 2017 ESC guidelines for the management of acute myocardial infarction in patients presenting with ST‐segment elevation: the task force for the management of acute myocardial infarction in patients presenting with ST‐segment elevation of the European Society of Cardiology (ESC). Eur Heart J. 2018;39(2):119‐177. 28886621 10.1093/eurheartj/ehx393

[15] Goodman SG , Nicolau JC , Requena G , et al. Longer‐term oral antiplatelet use in stable post‐myocardial infarction patients: insights from the long Term rIsk, clinical manaGement and healthcare Resource utilization of stable coronary artery dISease (TIGRIS) observational study. Int J Cardiol. 2017;236:54‐60. 28268087 10.1016/j.ijcard.2017.02.062

[16] Huo Y , Lee SW‐L , Sawhney JPS , et al. Two‐year outcomes post‐discharge in Asian patients with acute coronary syndrome: findings from the EPICOR Asia study. Int J Cardiol. 2020;5273(19):34212‐34213. 10.1016/j.ijcard.2020.05.022. 32389764

[17] Jan S , Lee SW , Sawhney JP , et al. Catastrophic health expenditure on acute coronary events in Asia: a prospective study. Bull World Health Organ. 2016;94(3):193‐200. 26966330 10.2471/BLT.15.158303 PMC4773930

[18] Jan S , Lee SW , Sawhney JPS , et al. Predictors of high‐cost hospitalization in the treatment of acute coronary syndrome in Asia: findings from EPICOR Asia. BMC Cardiovasc Disord. 2018;18(1):139. 29973147 10.1186/s12872-018-0859-4 PMC6033225

[19] Bueno H , Danchin N , Tafalla M , Bernaud C , Annemans L , van de Werf F . EPICOR (long‐tErm follow‐up of antithrombotic management Patterns In acute CORonary syndrome patients) study: rationale, design, and baseline characteristics. Am Heart J. 2013;165(1):8‐14. 23237128 10.1016/j.ahj.2012.10.018

[20] Pocock SJ , Huo Y , van de Werf F , et al. Predicting two‐year mortality from discharge after acute coronary syndrome: an internationally‐based risk score. Eur Heart J Acute Cardiovasc Care. 2019;8(8):727‐737. 28777005 10.1177/2048872617719638

[21] Hahn JY , Song YB , Oh JH , et al. 6‐month versus 12‐month or longer dual antiplatelet therapy after percutaneous coronary intervention in patients with acute coronary syndrome (SMART‐DATE): a randomised, open‐label, non‐inferiority trial. Lancet. 2018;391(10127):1274‐1284. 29544699 10.1016/S0140-6736(18)30493-8

[22] Rozemeijer R , Voskuil M , Greving JP , Bots ML , Doevendans PA , Stella PR . Short versus long duration of dual antiplatelet therapy following drug‐eluting stents: a meta‐analysis of randomised trials. Neth Heart J. 2018;26(5):242‐251. 29541996 10.1007/s12471-018-1104-6 PMC5910311

[23] Lee SY , Hong MK , Palmerini T , et al. Short‐term versus long‐term sual antiplatelet therapy after drug‐eluting stent implantation in elderly patients: a meta‐analysis of individual participant data from 6 randomized trials. JACC Cardiovasc Interv. 2018;11(5):435‐443. 29454730 10.1016/j.jcin.2017.10.015

[24] Udell JA , Bonaca MP , Collet JP , et al. Long‐term dual antiplatelet therapy for secondary prevention of cardiovascular events in the subgroup of patients with previous myocardial infarction: a collaborative meta‐analysis of randomized trials. Eur Heart J. 2016;37(4):390‐399. 26324537 10.1093/eurheartj/ehv443

[25] Pocock S , Bueno H , Licour M , et al. Predictors of one‐year mortality at hospital discharge after acute coronary syndromes: a new risk score from the EPICOR (long‐tErm follow uP of antithrombotic management patterns in acute CORonary syndrome patients) study. Eur Heart J Acute Cardiovasc Care. 2015;4(6):509‐517. 25301783 10.1177/2048872614554198 PMC4657391

[26] Chin CT , Ong TK , Krittayaphong R , et al. Characteristics and outcomes of medically managed patients with non‐ST‐segment elevation acute coronary syndromes: insights from the multinational EPICOR Asia study. Int J Cardiol. 2017;243:15‐20. 28747021 10.1016/j.ijcard.2017.04.059

[27] Wang L , Zhou Y , Qian C , Wang Y . Clinical characteristics and improvement of the guideline‐based management of acute myocardial infarction in China: a national retrospective analysis. Oncotarget. 2017;8(28):46540‐46548. 28147338 10.18632/oncotarget.14890 PMC5542290

[28] Bonaca MP , Bhatt DL , Cohen M , et al. Long‐term use of ticagrelor in patients with prior myocardial infarction. N Engl J Med. 2015;372(19):1791‐1800. 25773268 10.1056/NEJMoa1500857

[29] Steg PG , Bhatt DL , Simon T , et al. Ticagrelor in patients with stable coronary disease and diabetes. N Engl J Med. 2019;381(14):1309‐1320. 31475798 10.1056/NEJMoa1908077

[30] Bhatt DL , Steg PG , Mehta SR , et al. Ticagrelor in patients with diabetes and stable coronary artery disease with a history of previous percutaneous coronary intervention (THEMIS‐PCI): a phase 3, placebo‐controlled, randomised trial. Lancet. 2019;394(10204):1169‐1180. 31484629 10.1016/S0140-6736(19)31887-2

[31] D'Ascenzo F , Bertaina M , Fioravanti F , et al. Long versus short dual antiplatelet therapy in acute coronary syndrome patients treated with prasugrel or ticagrelor and coronary revascularization: insights from the RENAMI registry. Eur J Prev Cardiol. 2020;27(7):696‐705. 30862233 10.1177/2047487319836327

