# Supplementary material for: Long‐term antithrombotic management patterns in Asian patients with acute coronary syndrome: 2‐year observations from the EPICOR Asia study
Source: Clin Cardiol. 2020 Jul 2;43(9):999–1008. doi: 10.1002/clc.23400 (PMC7462192; doi:10.1002/clc.23400)
Supplement: Supplementary file 1 — SUPPLEMENTARY TABLE S1 Demographics, index diagnosis, and general health status of all patients discharged on DAPT who continued on DAPT for ≤12 vs >12 monthsa SUPPLEMENTARY TABLE S2 Demographics and baseline characteristics of patients discharged on SAPT/no antiplatelet or DAPTa SUPPLEMENTARY TABLE S3 Antithrombotic therapy at discharge and during follow‐up SUPPLEMENTARY TABLE S4 Logistic multivariable regression analysis for predictors of SAPT/no antiplatelet vs DAPTa at discharge SUPPLEMENTARY TABLE S5 Patients on continuous DAPT at each visit according to EPICOR 2‐year risk percentilesa [file CLC-43-999-s001.docx]

**Long-term antithrombotic management patterns in Asian patients with acute coronary syndrome: 2-year observations from the EPICOR Asia study**

SUPPORTING MATERIAL

SUPPLEMENTARY TABLE S1 Demographics, index diagnosis, and general health status of all patients discharged on DAPT who continued on DAPT for ≤12 vs >12 months^a^

|  | **Duration of DAPT following DAPT at discharge** | |  |
| --- | --- | --- | --- |
|  | **≤12 months  (n = 2364)** | **>12 months  (n = 9275)** | ***P*-value** |
| Duration of DAPT^a^, months; median (IQR) | 6.3 (2.7–11.2) | 23.6 (18.1–23.8) | <0.0001 |
| Age group |  |  | <0.0001 |
| ≤59 | 1022 (43.2) | 4564 (50.2) |  |
| 60–74 | 975 (41.2) | 3681 (39.7) |  |
| ≥75 | 367 (15.5) | 940 (10.1) |  |
| Male | 1757 (74.3) | 7249 (78.2) | <0.0001 |
| Final diagnosis of index event |  |  | 0.14 |
| STEMI | 1219 (51.6) | 4940 (53.3) |  |
| NSTE-ACS | 1145 (48.4) | 4335 (46.7) |  |
| BMI^b^, mean (SD) | 24.3 (3.5) | 24.8 (3.6) | <0.0001 |
| BMI^b^ |  |  | <0.001 |
| <25 (underweight / normal) | 1363 (61.4) | 4893 (56.9) |  |
| 25‒<30 kg/m^2^ (overweight) | 740 (33.4) | 3182 (37.0) |  |
| ≥30 kg/m^2^ (obese) | 116 (5.2) | 518 (6.0) |  |
| Killip class |  |  | <0.001 |
| I | 1660 (49.0) | 4976 (53.7) |  |
| II | 275 (11.6) | 983 (10.6) |  |
| III | 102 (4.3) | 319 (3.4) |  |
| IV | 73 (3.1) | 230 (2.5) |  |
| Missing | 754 (31.9) | 2767 (29.8) |  |
| Left bundle branch block | 33 (1.5) | 157 (1.8) | 0.36 |
| Ejection fraction |  |  | 0.04 |
| <30% | 44 (1.9) | 151 (1.6) |  |
| 30–40% | 146 (6.2) | 448 (4.8) |  |
| ≥40% | 1414 (59.8) | 5699 (61.4) |  |
| Missing | 760 (32.2) | 2977 (32.1) |  |
| Any CVD risk factors^b^ | 1564 (66.5) | 6072 (65.9) | 0.56 |
| Hypertension | 1274 (54.2) | 4859 (52.8) | 0.23 |
| Hypercholesterolemia | 398 (17.6) | 1617 (18.1) | 0.55 |
| Diabetes | 568 (24.3) | 2245 (24.5) | 0.81 |
| Family history of CAD | 206 (9.3) | 798 (9.1) | 0.73 |
| Current smoker | 795 (33.6) | 3700 (43.1) | 0.08 |
| Any prior CVD^b^ | 706 (30.3) | 2416 (26.7) | <0.001 |
| MI | 259 (11.2) | 828 (9.2) | 0.004 |
| Prior PCI | 196 (8.4) | 694 (7.7) | 0.22 |
| Prior CABG | 25 (1.1) | 121 (1.3) | 0.32 |
| CAG diagnostic for CAD | 201 (10.4) | 836 (9.3) | 0.09 |
| Angina | 363 (15.6) | 1410 (15.6) | 0.97 |
| Heart failure | 83 (3.6) | 194 (2.2) | <0.0001 |
| Atrial fibrillation | 49 (2.1) | 103 (1.1) | <0.001 |
| TIA/stroke | 114 (4.9) | 386 (4.3) | 0.17 |
| PVD | 22 (1.0) | 70 (0.8) | 0.40 |
| Chronic renal failure | 67 (2.9) | 122 (1.4) | <0.0001 |
| Any CV medication^b^ | 880 (38.7) | 3231 (36.5) | 0.06 |
| Any antiplatelet | 605 (26.7) | 2097 (23.9) | 0.004 |
| Aspirin | 579 (25.6) | 2028 (23.1) | 0.012 |
| Clopidogrel | 226 (10.0) | 805 (9.2) | 0.22 |
| β-blocker | 371 (17.2) | 1327 (15.4) | 0.05 |
| ACEi/ARB | 354 (16.4) | 1335 (15.5) | 0.34 |
| Statin | 358 (16.5) | 1255 (14.6) | 0.025 |
| In-hospital procedures^b^ |  |  |  |
| PCI/CABG | 1444 (61.8) | 7127 (78.1) | <0.0001 |
| Reperfusion (primary PCI or fibrinolysis) | 1547 (66.3) | 7445 (81.5) | <0.0001 |
| Any drug-eluting stent | 1227 (51.9) | 6381 (68.8) | <0.0001 |
| In-hospital MI/recurrent ischemia/heart failure/cardiogenic shock/arrhythmia^b^ | 427 (18.3) | 1179 (12.9) | <0.0001 |
| Country group^c^ |  |  | <0.0001 |
| China (n = 7525) | 1575 (66.6/20.9) | 5950 (64.2/79.1) |  |
| India (n = 2019) | 327 (13.8/16.2) | 1692 (18.2/83.8) |  |
| South Korea, Hong Kong, and Singapore (n = 902) | 184 (7.8/20.4) | 718 (7.7/79.6) |  |
| Malaysia, Thailand, and Vietnam (n = 1193) | 278 (11.8/23.3) | 915 (9.9/76.7) |  |
| Time from symptom onset to admission, hours; median (IQR) | 5.5 (2.2–18.0) | 5.7 (2.3–18.0) | 0.44 |
| Time from admission to reperfusion, hours; median (IQR) | 13.2 (1.3–96.1) | 14.0 (1.4–91.9) | 0.77 |
| Time from symptom onset to reperfusion, hours; median (IQR) | 24.0 (4.8–104.8) | 27.1 (5.7–12.0) | 0.03 |
| Length of hospital stay, days; median (IQR) | 9.0 (6.0–13.0) | 8.0 (5.0–12.0) | 0.002 |
| Dependence at discharge |  |  | 0.07 |
| No dependence | 2224 (94.1) | 8698 (93.8) |  |
| Non-severe dependence | 122 (5.2) | 537 (5.8) |  |
| Severe dependence | 18 (0.8) | 40 (0.4) |  |
| EQ-5D overall health state at discharge, mean (SD) | 77.9 (14.1) | 78.9 (13.8) | <0.001 |

Abbreviations: ACEi/ARB, angiotensin-converting enzyme inhibitor/angiotensin II receptor blocker; BMI, body mass index; CABG, coronary artery bypass graft; CAD, coronary artery disease; CAG, coronary angiogram; CHD, coronary heart disease; CV, cardiovascular; CVD, cardiovascular disease; DAPT, dual antiplatelet therapy; EQ-5D, EuroQol-5 Dimensions; IQR, interquartile range; MI, myocardial infarction; MI, myocardial infarction; NSTE-ACS, non-ST elevation acute coronary syndrome; STEMI, ST-elevation myocardial infarction; PCI, percutaneous coronary intervention; PVD, peripheral vascular disease; TIA, transient ischemic attack. Values are n (%) unless otherwise indicated.

^a^Includes patients reported as taking two or more antiplatelets at discharge (ie, including triple antiplatelet therapy). DAPT duration was defined as time from discharge to the last use of two or more antiplatelets, not accounting for interruptions.

^b^At discharge, data were missing for 879 patients for BMI, 68 patients for any CVD risk factors, 244 patients for any prior CVD, 562 patients for any CV medication, 172 patients for in-hospital PCI/CABG, 169 patients for primary PCI/fibrinolysis and 128 patients for in-hospital MI/recurrent ischemia/heart failure/cardiogenic shock/arrhythmia.

^c^Percentages shown are within DAPT duration group/within each country/region.

^a^Includes patients reported as taking two or more antiplatelets (ie, including triple antiplatelet therapy).

# SUPPLEMENTARY TABLE S2 Demographics and baseline characteristics of patients discharged on SAPT/no antiplatelet or DAPT^a^

|  | **SAPT/none at discharge (n = 1283)** | **DAPT at discharge (n = 11 639)** | ***P*-value** |
| --- | --- | --- | --- |
| Age group, years |  |  | <0.0001 |
| ≤59 | 535 (41.7) | 5676 (48.8) |  |
| 60–74 | 541 (42.2) | 4656 (40.0) |  |
| ≥75 | 207 (16.1) | 1307 (11.2) |  |
| Male | 851 (66.3) | 9006 (77.4) | <0.0001 |
| Final diagnosis of index event |  |  | <0.0001 |
| STEMI | 457 (35.6) | 6159 (52.9) |  |
| NSTE-ACS | 826 (64.4) | 5480 (47.1) |  |
| BMI^b^ |  |  | <0.05 |
| <25 (underweight/normal) | 632 (60.2) | 6256 (57.9) |  |
| 25‒<30 kg/m^2^ (overweight) | 343 (32.7) | 3922 (36.3) |  |
| ≥30 kg/m^2^ (obese) | 75 (7.1) | 634 (5.9) |  |
| Any CVD risk factors^b^ | 853 (67.0) | 7636 (66.0) | 0.47 |
| Hypertension | 710 (55.8) | 6133 (53.1) |  |
| Hypercholesterolemia | 183 (14.9) | 2015 (18.0) |  |
| Diabetes | 349 (27.6) | 2813 (24.5) |  |
| Family history of CAD | 84 (7.5) | 1004 (9.2) |  |
| Current smoker | 288 (22.5) | 4091 (35.2) |  |
| Any prior CVD^b^ | 397 (32.0) | 3122 (27.4) | <0.001 |
| MI | 142 (11.5) | 1087 (9.6) | 0.03 |
| Prior PCI | 88 (7.1) | 890 (7.8) | 0.38 |
| Prior CABG | 28 (2.3) | 146 (1.3) | <0.01 |
| CAG diagnostic for CAD | 145 (11.8) | 1077 (9.5) | 0.01 |
| Angina | 203 (16.4) | 1773 (15.6) | 0.45 |
| Heart failure | 43 (3.5) | 277 (2.5) | <0.05 |
| Atrial fibrillation | 36 (2.9) | 152 (1.3) | <0.0001 |
| TIA/stroke | 57 (5.4) | 500 (4.4) | 0.10 |
| PVD | 10 (0.8) | 92 (0.8) | 0.99 |
| Chronic renal failure | 34 (2.7) | 189 (1.7) | <0.01 |
| Any CV medication^b^ | 441 (39.3) | 4111 (36.9) | 0.11 |
| Any antiplatelet | 279 (25.2) | 2702 (24.4) | 0.57 |
| Aspirin | 247 (22.3) | 2607 (23.6) | 0.34 |
| Clopidogrel | 98 (8.9) | 1031 (9.3) | 0.60 |
| β-blocker | 192 (18.2) | 1698 (15.8) | <0.05 |
| ACEi/ARB | 185 (17.5) | 1689 (15.7) | 0.13 |
| Statin | 181 (17.0) | 1613 (15.0) | 0.08 |
| In-hospital procedures^b^ |  |  |  |
| PCI/CABG | 279 (22.9) | 8571 (74.7) | <0.0001 |
| Reperfusion (primary PCI or fibrinolysis) | 324 (26.5) | 8992 (78.4) | <0.0001 |
| Any drug-eluting stent | 189 (14.7) | 7608 (65.4) | <0.0001 |
| In-hospital MI/recurrent ischemia/heart failure/cardiogenic shock/arrhythmia^b^ | 166 (13.3) | 1606 (14.0) | 0.56 |
| Number of antiplatelets at discharge |  |  | <0.0001 |
| 0 | 162 (12.6) | 0 |  |
| 1 | 1121 (87.4) | 0 |  |
| ≥2 | 0 | 11,639 (100) |  |
| Country group^c^ |  |  | <0.0001 |
| China | 689 (8.4) | 7525 (91.6) |  |
| India | 449 (18.2) | 2019 (81.8) |  |
| South Korea, Hong Kong, and Singapore | 73 (7.5) | 902 (92.5) |  |
| Malaysia, Thailand, and Vietnam | 72 (5.7) | 1193 (94.3) |  |
| Country^c^ |  |  | <0.0001 |
| China | 689 (8.4) | 7525 (91.6) |  |
| Hong Kong | 23 (13.0) | 154 (87.0) |  |
| India | 449 (18.2) | 2019 (81.8) |  |
| Malaysia | 11 (11.0) | 89 (89.0) |  |
| Singapore | 10 (10.8) | 83 (89.2) |  |
| South Korea | 40 (5.7) | 665 (94.3) |  |
| Thailand | 55 (5.7) | 902 (94.3) |  |
| Vietnam | 6 (2.9) | 202 (97.1) |  |
| Residency |  |  | 0.19 |
| Rural | 448 (34.9) | 4283 (36.8) |  |
| Metropolitan | 835 (65.1) | 7356 (63.2) |  |
| Insurance |  |  |  |
| Government | 758 (59.1) | 8283 (71.2) | <0.0001 |
| Private | 137 (10.7) | 1196 (10.3) | 0.65 |
| Employer provided | 23 (1.8) | 191 (1.6) | 0.69 |
| Other | 31 (2.4) | 463 (4.0) | <0.01 |
| None | 355 (27.7) | 1724 (14.8) | <0.0001 |
| Education^b^ |  |  | <0.0001 |
| No formal education | 119 (12.2) | 929 (9.4) |  |
| Primary | 223 (22.8) | 2751 (27.7) |  |
| Secondary | 392 (40.1) | 4347 (43.8) |  |
| University |  |  |  |
| Professional status^b^ |  |  | <0.0001 |
| Employed/has own business | 451 (41.8) | 5691 (53.4) |  |
| Unemployed | 64 (5.9) | 557 (5.2) |  |
| Homemaker | 97 (9.0) | 726 (6.8) |  |
| Retired | 445 (41.3) | 3487 (32.7) |  |
| Student | 2 (0.2) | 9 (0.1) |  |
| Sick leave | 14 (1.3) | 125 (1.2) |  |
| Maternity leave | 2 (0.2) | 14 (0.1) |  |
| Disability pension | 3 (0.3) | 41 (0.4) |  |
| Time from symptom onset to admission, hours; median (IQR)^b^ | 6.7 (2.0–23.8) | 5.7 (2.3–18.0) | 0.01 |
| Time from admission to reperfusion, hours; median (IQR)^b,d^ | 3.5 (1.0–35.8) | 13.9 (1.4–92.5) | <0.0001 |
| Time from symptom onset to reperfusion, hours; median (IQR)^b,d^ | 17.0 (4.5–49.9) | 26.3 (5.5–104.5) | <0.0001 |
| Length of hospital stay, d; median (IQR) | 8.0 (5.0–12.0) | 9.0 (5.0–13.0) | <0.0001 |
| Killip class |  |  | <0.0001 |
| I | 455 (35.5) | 6136 (52.7) |  |
| II | 135 (10.5) | 1258 (10.8) |  |
| III | 53 (4.1) | 421 (3.6) |  |
| IV | 25 (2.0) | 303 (2.6) |  |
| Missing | 615 (47.9) | 3521 (30.3) |  |
| Left bundle branch block^b^ | 21 (1.8) | 190 (1.7) | 0.88 |
| Positive cardiac markers^b^ | 710 (57.4) | 8465 (73.8) | <0.0001 |
| Ejection fraction |  |  | <0.0001 |
| <30% | 30 (2.3) | 195 (1.7) |  |
| 30–40% | 50 (3.9) | 594 (5.1) |  |
| ≥40% | 562 (43.8) | 7113 (61.1) |  |
| Missing | 641 (50.0) | 3737 (32.1) |  |
| Laboratory values, median (IQR)^b^ |  |  |  |
| White blood cell count/mL | 7895 (6120–10 300) | 8830 (6800–11 420) | <0.0001 |
| Initial creatinine level, mg/dL | 0.90 (0.77–1.10) | 0.90 (0.76–1.08) | 0.33 |
| ≥2 mg/dL, n (%) | 215 (8.3) | 1892 (16.9) | 0.20 |
| Glucose level, mg/dL | 113.9 (95.0–146.0) | 120.0 (98.7–157.5) | <0.0001 |
| Hemoglobin level, g/dL | 13.4 (12.0–14.6) | 13.8 (12.5–15.0) | <0.0001 |
| Dependence at discharge |  |  | <0.0001 |
| No dependence | 1166 (90.9) | 10,922 (93.8) |  |
| Non-severe dependence | 103 (8.0) | 659 (5.7) |  |
| Severe dependence | 14 (1.1) | 58 (0.5) |  |
| EQ-5D overall score (health state) at discharge, mean (SD)^b^ | 77.2 (13.8) | 78.7 (13.9) | <0.001 |
| EQ-5D simple score^b^ |  |  | <0.0001 |
| 0 | 745 (58.4) | 7580 (65.2) |  |
| 1 | 172 (13.5) | 1383 (11.9) |  |
| ≥2 | 359 (28.1) | 2669 (23.0) |  |

Abbreviations: ACEi/ARB, angiotensin-converting enzyme inhibitor/angiotensin II receptor blocker; BMI, body mass index; CABG, coronary artery bypass graft; CAD, coronary artery disease; CAG, coronary angiogram; CHD, coronary heart disease; CV, cardiovascular; CVD, cardiovascular disease; DAPT, dual antiplatelet therapy; EQ-5D, EuroQol Five Dimensions; IQR, interquartile range; MI, myocardial infarction; NSTE-ACS, non-ST elevation acute coronary syndrome; PCI, percutaneous coronary intervention; PVD, peripheral vascular disease; SAPT, single antiplatelet therapy; STEMI, ST-elevation myocardial infarction; TIA, transient ischemic attack. Values are n (%) unless otherwise indicated.

^a^Includes patients reported as taking two or more antiplatelets at discharge (ie, including triple antiplatelet therapy).

^b^Data were missing for 233 SAPT/none and 879 DAPT patients for BMI, 10 SAPT/none and 68 DAPT patients for any CVD risk factors, 41 SAPT/none and 244 DAPT patients for any prior CVD, 162 SAPT/none and 562 DAPT patients for any CV medication, 63 SAPT/none and 172 DAPT patients for in-hospital PCI/CABG, 60 SAPT/none and 169 DAPT patients for primary PCI/fibrinolysis, 39 SAPT/none and 128 DAPT patients for in-hospital MI/recurrent ischemia/heart failure/cardiogenic shock/arrhythmia, 306 SAPT/none and 1717 DAPT patients for education, 205 SAPT/none and 989 DAPT patients for professional status, 306 SAPT/none and 1530 DAPT patients for time from symptom onset to admission, 1091 SAPT/none and 4205 DAPT patients for time from admission to reperfusion, 1010 SAPT/none and 2772 DAPT patients for time from symptom onset to reperfusion, 112 SAPT/none and 675 DAPT patients for left bundle branch block, 46 SAPT/none and 165 DAPT patients for positive cardiac markers, 135 SAPT/none and 666 DAPT patients for white blood cell count, 110 SAPT/none and 411 DAPT patients for initial creatinine, 205 SAPT/none and 1276 DAPT patients for glucose, 105 SAPT/none and 484 DAPT patients for hemoglobin, and 7 SAPT/none and 21 DAPT patients for EQ-5D overall and simple score.

^c^Percentages shown are within each country.

^d^Includes intervention and fibrinolysis.

# SUPPLEMENTARY TABLE S3 Antithrombotic therapy at discharge and during follow-up

|  | **Discharge**  **(n = 12,784)** | **Time post-discharge (months)** | | | | | | |
| --- | --- | --- | --- | --- | --- | --- | --- | --- |
|  |  | **1.5  (n = 12,588)** | **3  (n = 12,424)** | **6  (n = 12,245)** | **9  (n = 12,035)** | **12  (n = 11,776)** | **18  (n = 11,198)** | **23  (n = 10,422)** |
| Antiplatelet (AP) | | | | | | | | |
| None | 23 (0.2) | 89 (0.7) | 132 (1.1) | 208 (1.7) | 270 (2.2) | 347 (3.0) | 541 (4.8) | 602 (5.8) |
| Aspirin only | 672 (5.3) | 768 (6.1) | 894 (7.2) | 1050 (8.6) | 1117 (9.3) | 1547 (13.1) | 3059 (27.3) | 3250 (31.2) |
| ADPra only | 447 (3.5) | 480 (3.8) | 497 (4.0) | 508 (4.2) | 532 (4.4) | 565 (4.8) | 588 (5.3) | 567 (5.4) |
| Other SAPT | 2 (0.02) | 3 (0.02) | 4 (0.03) | 6 (0.05) | 8 (0.07) | 11 (0.09) | 11 (0.10) | 16 (0.15) |
| Aspirin + ADPra | 11,348 (88.8) | 10,998 (87.4) | 10,655 (85.8) | 10,268 (83.9) | 9923 (82.5) | 9129 (77.5) | 6842 (61.1) | 5844 (56.1) |
| Other DAPT | 69 (0.5) | 65 (0.5) | 65 (0.5) | 65 (0.5) | 63 (0.5) | 60 (0.5) | 60 (0.5) | 60 (0.6) |
| TAPT | 222 (1.7) | 184 (1.5) | 176 (1.4) | 139 (1.1) | 121 (1.0) | 116 (1.0) | 96 (0.9) | 82 (0.8) |
| Anticoagulant (AC) | | | | | | | | |
| AC only | 10 (0.08) | 9 (0.07) | 10 (0.08) | 9 (0.07) | 9 (0.07) | 12 (0.10) | 14 (0.13) | 12 (0.12) |
| AC with AP | 127 (0.99) | 117 (0.93) | 112 (0.90) | 110 (0.90) | 107 (0.89) | 101 (0.86) | 96 (0.86) | 88 (0.84) |
| Death or lost to follow-up | | | | | | | | |
| Death | – | 91 | 159 | 245 | 330 | 416 | 553 | 638 |
| Lost to follow-up | – | 105 | 201 | 294 | 419 | 592 | 1033 | 1722 |

Abbreviations: AC, anticoagulant; ADPra, adenosine diphosphate receptor antagonist; AP, antiplatelet; DAPT, dual antiplatelet therapy; SAPT, single antiplatelet therapy; TAPT, triple antiplatelet therapy. Values are n (%).

SUPPLEMENTARY TABLE S4. Logistic multivariable regression analysis for predictors of SAPT/no antiplatelet versus DAPT^a^ at discharge

| **Factor** | **OR^b^** | **95% CI** | ***P*-value** |
| --- | --- | --- | --- |
| Anticoagulant at discharge, vs no | 19.73 | 13.18, 29.55 | <0.0001 |
| Any drug-eluting stent, vs no | 0.26 | 0.19, 0.34 | <0.0001 |
| Dependence^c^ at discharge, vs no dependence |  |  | 0.0080 |
| Non-severe | 1.44 | 1.11, 1.86 |  |
| Severe | 1.85 | 0.89, 3.85 |  |
| Ejection fraction, vs <30% |  |  | <0.0001 |
| 30‒40% | 0.59 | 0.33, 1.06 |  |
| ≥40% | 0.67 | 0.41, 1.10 |  |
| Missing | 1.07 | 0.65, 1.76 |  |
| Killip class at admission, vs class I |  |  | <0.0001 |
| II | 1.20 | 0.95, 1.52 |  |
| III | 0.91 | 0.63, 1.33 |  |
| IV | 1.18 | 0.71, 1.97 |  |
| Missing | 2.11 | 1.80, 2.47 |  |
| In-hospital PCI/CABG, vs no | 0.32 | 0.25, 0.41 | <0.0001 |
| Female, vs male | 1.44 | 1.24, 1.67 | <0.0001 |
| Country group, vs China |  |  | <0.0001 |
| India | 1.55 | 1.30, 1.83 |  |
| South Korea, Hong Kong, and Singapore | 0.99 | 0.73, 1.34 |  |
| Malaysia, Thailand, and Vietnam | 0.41 | 0.31, 0.54 |  |

Abbreviations: CABG, coronary artery bypass graft; CI, confidence interval; DAPT, dual antiplatelet therapy; OR, odds ratio; PCI, percutaneous coronary intervention; SAPT, single antiplatelet therapy.

^a^Stepwise selection procedure using logistic regression model for likelihood of SAPT/No antiplatelet (vs DAPT) at discharge from the index hospitalization.

^b^OR >1 indicates greater likelihood of SAPT/no antiplatelet therapy at discharge.

^c^Investigator determined.

# SUPPLEMENTARY TABLE S5 Patients on continuous DAPT at each visit according to EPICOR 2-year risk percentiles^a^

|  | **EPICOR 2-year risk score percentiles (median score = 3.403)** | | | | | | | |
| --- | --- | --- | --- | --- | --- | --- | --- | --- |
|  | **≤60th (≤3.646; n = 7193)** | | **>60th to ≤80th (>3.646–4.252; n = 2271)** | | **>80th to ≤90th (>4.252–4.796; n = 1102)** | | **>90th (>4.796; n = 1073)** | |
| **Visit** | **Continuous DAPT** | **Lost to follow-up** | **Continuous DAPT** | **Lost to follow-up** | **Continuous DAPT** | **Lost to follow-up** | **Continuous DAPT** | **Lost to follow-up** |
| 6 weeks | 7025 (97.7) | 45 (0.6) | 2193 (96.6) | 36 (1.6) | 1042 (94.6) | 29 (2.6) | 982 (91.5) | 62 (5.8) |
| 3 months | 6829 (94.9) | 98 (1.4) | 2132 (93.9) | 61 (2.7) | 1011 (91.7) | 42 (3.8) | 915 (85.3) | 110 (10.3) |
| 6 months | 6643 (92.4) | 151 (2.1) | 2022 (89.0) | 100 (4.4) | 947 (85.9) | 62 (5.6) | 851 (79.3) | 154 (14.4) |
| 9 months | 6437 (89.5) | 249 (3.5) | 1958 (86.2) | 130 (5.7) | 911 (82.7) | 79 (7.2) | 790 (73.6) | 198 (18.5) |
| 12 months | 5927 (82.4) | 363 (5.0) | 1814 (79.9) | 171 (7.5) | 841 (76.3) | 111 (10.1) | 713 (66.5) | 240 (22.4) |
| 15 months | 4927 (68.5) | 494 (6.9) | 1536 (67.6) | 229 (10.1) | 742 (67.3) | 142 (12.9) | 632 (58.9) | 275 (25.6) |
| 18 months | 4338 (60.3) | 641 (8.9) | 1385 (61.0) | 282 (12.4) | 680 (61.7) | 177 (16.1) | 585 (54.5) | 299 (27.9) |
| 21 months | 3891 (54.1) | 890 (12.4) | 1256 (55.3) | 367 (16.2) | 630 (57.2) | 219 (19.9) | 528 (49.2) | 348 (32.4) |
| 23 months | 3697 (51.4) | 1029 (14.3) | 1184 (52.1) | 424 (18.7) | 594 (53.9) | 251 (22.8) | 502 (46.8) | 377 (35.1) |

Abbreviations: DAPT, dual antiplatelet therapy; EPICOR, long-tErm follow-up of antithrombotic management Patterns In acute CORonary syndrome.

^a^Includes patients reported as taking two or more antiplatelets (ie, including triple antiplatelet therapy).
